# Itch Is Required for Lateral Line Development in Zebrafish

**DOI:** 10.1371/journal.pone.0111799

**Published:** 2014-11-04

**Authors:** Annie Angers, Pierre Drapeau

**Affiliations:** 1 Department of Biological Sciences, University of Montreal, Montreal, Quebec, Canada; 2 Department of Neuroscience and Research Centre of the Hospital Centre of the University of Montreal, Montreal, Quebec, Canada; 3 GRSNC, University of Montreal, Montreal, Quebec, Canada; Baylor Institute for Immunology Research, United States of America

## Abstract

The zebrafish posterior lateral line is formed during early development by the deposition of neuromasts from a migrating primordium. The molecular mechanisms regulating the regional organization and migration of the primordium involve interactions between Fgf and Wnt/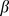-catenin signaling and the establishment of specific *cxcr4b* and *cxcr7b* cytokine receptor expression domains. Itch has been identified as a regulator in several different signaling pathways, including Wnt and Cxcr4 signaling. We identified two homologous *itch* genes in zebrafish, *itcha* and *itchb*, with generalized expression patterns. By reducing *itchb* expression in particular upon morpholino knockdown, we demonstrated the importance of Itch in regulating lateral line development by perturbing the patterns of *cxcr4b* and *cxcr7b* expression. Itch knockdown results in a failure to down-regulate Wnt signaling and overexpression of *cxcr4b* in the primordium, slowing migration of the posterior lateral line primordium and resulting in abnormal development of the lateral line.

## Introduction

The zebrafish lateral line is an insightful system for studies of cellular development as it displays evolutionarily-conserved developmental mechanisms ranging from progenitor migration, neural differentiation and planar cell polarity to sensory transduction [1]. Furthermore, the lateral line is thought to have evolved into sensory structures of the cochlea and inner ear in drier vertebrates, making it an organ of broad interest for developmental neurobiology [2], [3]. The posterior lateral line (pLL) is a mechanosensory organ running along the body and tail of fish and amphibians. It is built during early development through coordinated cell migration, proliferation, epithelial morphogenesis and differentiation of a group of about one hundred cells forming the pLL primordium. The pLL primordium arises from placodal cells that undergo partial epithelial-mesenchymal transition and acquire migratory properties. As the primordium migrates towards the tail along the myoseptum, cells in the trailing zone of the primordium become organized into rosette-like epithelial structures that mature into proneuromasts, which are reiteratively formed and deposited every 3–4 hours. These cells differentiate as the accessory and hair cells of the 6–7 mature neuromasts of the primary pLL. When the primordium reaches the end of the tail, it fragments into a few terminal neuromasts [4]–. Thus, the timing of neuromast deposition and the underlying molecular mechanisms of its regulation are critical for the development of this organ.

The migration of the primordium and the formation of the neuromasts is coordinated by Wnt and Fgf signaling. Through a feedback mechanism, Wnt/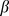-catenin signaling is restricted to the leading zone of the primordium and Fgf signaling occurs in the trailing zone [7], [8]. These localized activities maintain the polarized activation of two chemokine receptors: *cxcr4b* is expressed in the leading zone while *cxcr7b* is restricted to the trailing zone. The differential expression of *cxcr4b* and *cxcr7b* is essential for directed collective migration of the primordium cells [1], [9]. However, the mechanisms downstream of these receptors that convey their actions are unclear.

The ubiquitin ligase ITCH has been shown to influence signaling downstream of several important receptors. In particular, ITCH recognizes and down-regulates several SH3-domain proteins, which have been shown to limit epidermal growth factor receptor internalization and signaling [10]. Although no direct link has been established between ITCH and FGF signaling, ITCH targets proteins involved in receptor tyrosine kinase internalization like CBL and SH3GL2 (endophilin) [10]–[12].

ITCH directly interacts with ligand-activated CXCR4 and promotes its ubiquitylation at the plasma membrane [13], [14], which is important for the regulation of CXCR4 trafficking and signaling [14], [15]. In human cell lines, ITCH depletion significantly attenuates CXCR4-induced ERK-1/2 activation and modestly increases CXCR4 surface levels [16].

ITCH also regulates Wnt signaling through its interaction with Disheveled (Dvl) [17]. Dvl is a central mediator of Wnt signaling where it functions as a scaffold protein bridging the receptors and downstream signaling components [18], [19]. In HEK-293 cells, knockdown of ITCH significantly increased Wnt-induced TOPflash activity and the accumulation of free 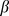-catenin induced by Wnt3a. Wnt3a-mediated induction of Wnt target genes *AXIN2* and *NKD1* was also potentiated, suggesting that ITCH negatively regulates the canonical Wnt pathway [17].

Given this implication of Itch in the two major signaling pathways, Cxcr4 and Wnt, involved in pLL primordium migration, we investigated the effects of Itch depletion in lateral line formation in zebrafish embryos. Our study presents the first direct demonstration of the implication of the ubiquitin-ligase Itch in the regulation of signal transduction in a living organism.

## Results

### Itch in *Danio rerio*

To investigate the impact of *itch* loss of function in early vertebrate development, we used the well-established model that is the zebrafish [20]. We identified the *ITCH* orthologues in zebrafish by querying the NCBI database *Danio rerio* sequences with the amino acid sequence of the human Itch HECT domain (NP_113671.3) using the tBlastn algorithm [21]. This search retrieved 11 complete coding sequences with a maximum nucleotide sequence identity of 43–88%. All the sequences identified encoded predicted proteins with the typical domain architecture of the Nedd4 sub-family of ubiquitin ligases, consisting of a C2 domain, two to four WW domains and the catalytic HECT domain (CWH ligases).

**Figure 1:**
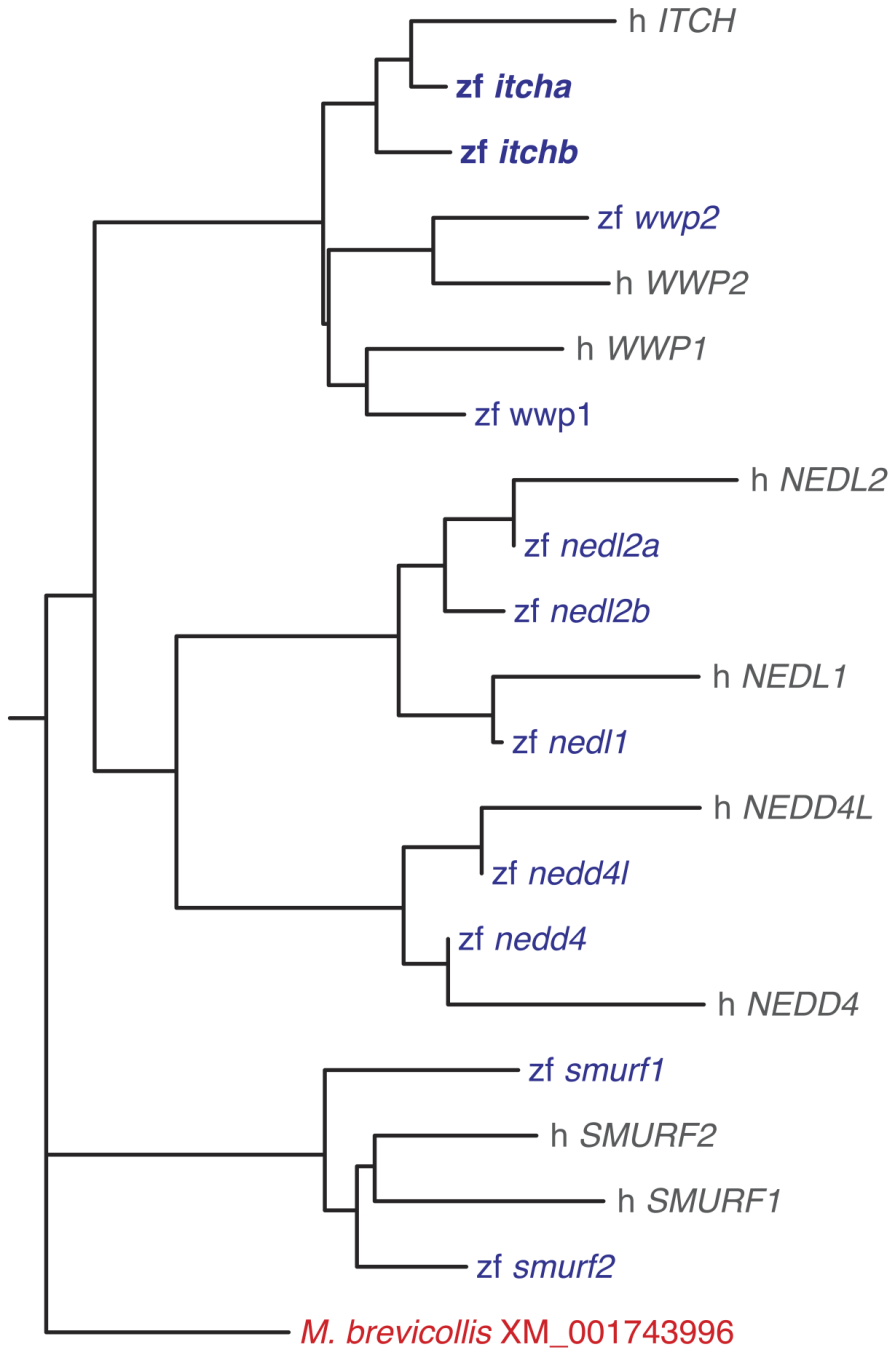
Phylogenetic tree of human and zebrafish members of the CWH family of E3s. Two zebrafish sequences cluster with the human *ITCH* gene. The tree was constructed with the neighbor-joining method supported by 1000 bootstraps. Sequences used in the phylogenetic analysis are given in Table S1.

We aligned these *cds* sequences together with the human sequences for all nine members of the CWH sub-family (Table S1), and constructed a phylogenetic tree using the Phylip package SeqDist algorithm (Fig. 1) [22]. All zebrafish sequences clustered with their human counterparts, giving weight to the hypothesis that they represent orthologues. Two genes grouped with human *ITCH*. We designate these sequences *itcha* and *itchb*, corresponding respectively to NCBI gene IDs 100331274 on chromosome 6 and 100330031 on chromosome 23. Interestingly, the *itchb* sequence is absent from the ZFIN database but was however confirmed in the latest zebrafish genome sequencing project [23]. Except for *itch* and *nedl2*, only one copy of each of the other CWHs was found in the zebrafish genome.

**Figure 2:**
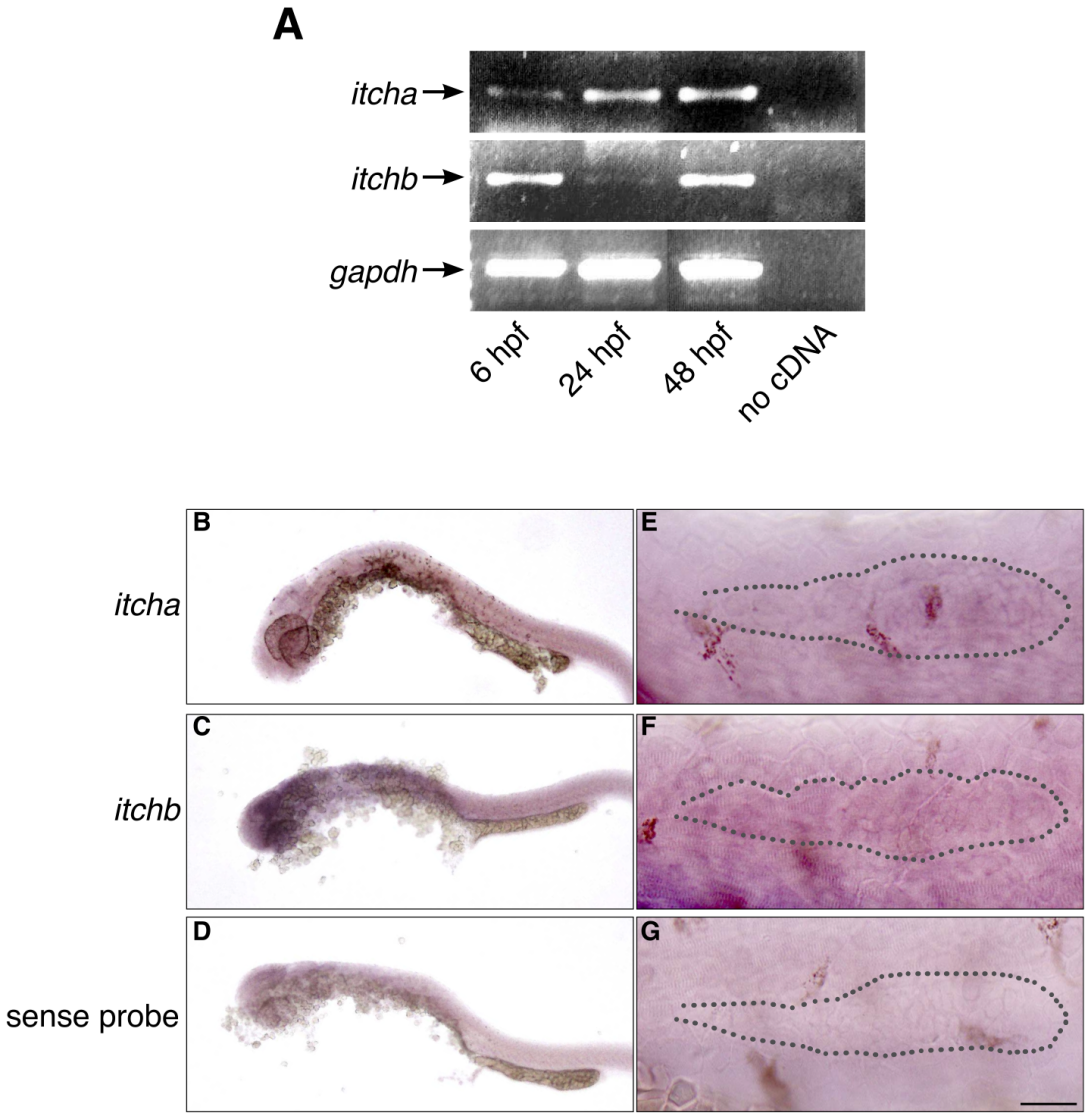
Expression of *itch* genes in zebrafish embryos. (A) RT-PCR experiment showing early expression of both *itcha* and *itchb* genes. RNA was extracted from embryos at approximately 6, 24 and 48 hpf. cDNA was obtained from each stage using 1 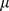
g total RNA. *GAPDH* was used as an internal control for cDNA amplification. (B–D) Weak, general staining of embryos at 26 hpf revealed with DIG-labelled probes complementary to *itcha* (B) or *itchb* (C) or sense sequence (D) as a control. (E–G) The primordium of 26 hpf embryos revealed with DIG-labelled probes complementary to *itcha* (E) or *itchb* (F) or sense sequence (G) as a control. Position of the primordium is underlined with a dotted line. Expression of *itch* genes is general, and *itchb* is expressed throughout the primordium. Scale bar: (B–D) 250 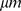, (E–G) 25 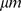.

We next examined the expression pattern of *itcha* and *itchb* in developing embryos. We preformed reverse-transcription of RNA extracted from embryos at 6, 24, and 48 hours post-fertilization (hpf) followed by PCR amplification with primers designed to specifically amplify *itcha* or *itchb*. Both genes were successfully amplified from 6 hpf embryos and their expression was maintained at 48 hpf (Fig. 2 A) and later on (not shown). *In situ* hybridization experiments were conducted on embryos at 6 and 24 hpf. A generalized pattern of expression was discerned in these experiments, suggesting that both genes are expressed in every tissue (Fig. 2 B–D). This is consistent with reports *ITCH* gene in mammals where RNA is detected in all analyzed tissues [24]. Faint staining could be observed in the pLL primordium with both *itcha* and *itchb* specific probes, distributed throughout the structure, whereas no staining appeared when sense probes were used (Fig. 2 E–G).

### Knockdown of *itcha* or *itchb* affects embryo's growth

**Figure 3:**
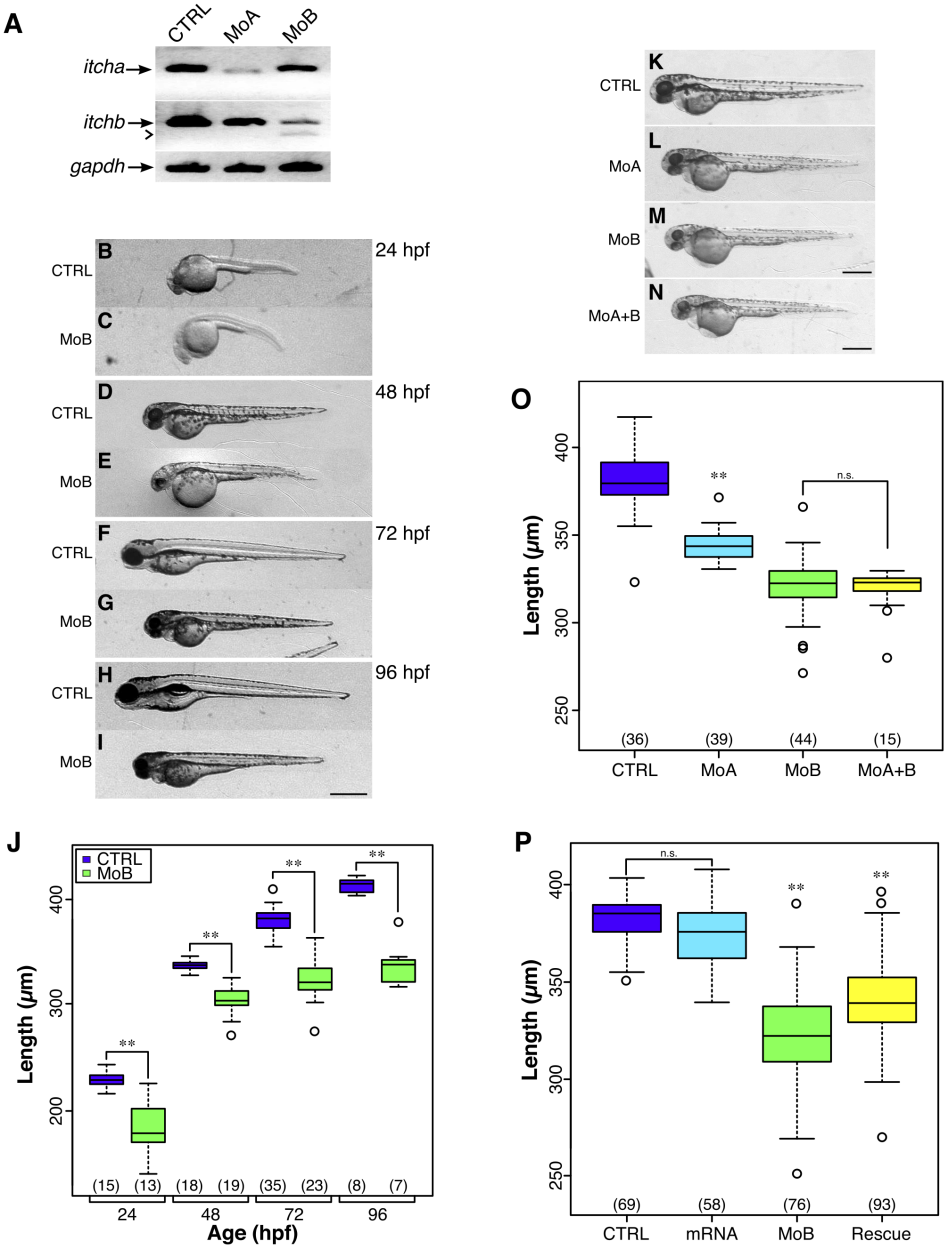
*itch* knockdown impairs early zebrafish development. (A) Splice junction MOs against *itcha* and *itchb* efficiently reduced their respective but not opposite mRNAs. RNA was extracted from 48 hpf control embryos or embryos injected with MOs against *itcha* (MoA) or *itchb* (MoB). After reverse-transcription, cDNAs from each group were amplified with PCR primers specific for *itcha*, *itchb* or GAPDH as a control for 35 cycles. Marked reduction of *itcha* and *itchb* was obtained after injection of the appropriate but not the other MO. (B–I) Morphology of embryos injected with MOs targeting *itchb* (MoB) showed general growth failure, already visible at 24 hpf (B,C) and persisting through to 96 hpf (H,I) as compared to control embryos (CTRL). Scale bar: 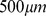. (J) Total length of control embryos or embryos injected with MOs against *itchb* (MoB) was measured from the tip of the head to the tip of the tail muscle. Two-factor analysis of variance indicates no significant interaction of injection with age. Pairwise comparison was performed between control and MoB injected embryos at each time point. The number of embryos measured is indicated at the bottom of the graph. 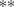 represents statistical significance at 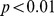. (K-N) Morphology of control embryos (CTRL), *itcha* knockdown embryos (MoA), *itchb* knockdown (MoB) or *itcha* and *itchb* knockdown embryos (MoA
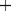
B) at 48 hpf. *itcha* knockdown embryos were slightly delayed, but were morphologically intact as compared to *itchb* or *itcha* plus *itchb* knockdown embryos. (O) Global growth of injected embryos was assessed by total length measurements at 72 hpf. *itcha* and *itchb* knockdown embryos were significantly smaller than control embryos, and *itchb* knockdown were smaller than *itcha* as assessed by Kruskal-Wallis one-way ANOVA combined with Dunn's method of group comparison with a significant threshold fixed at 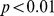 (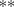). *itcha* plus *itchb* knockdown embryos were not significantly smaller than *itchb* knockdown embryos (n.s.). (P) Growth defect of *itchb* knockdown was partially rescued with injection of *in vitro* transcribed human *ITCH* mRNA. Kruskal-Wallis one-way ANOVA combined with Dunn's method of comparison established no-significant differences between control embryos (CTRL) and embryos injected with *ITCH* mRNA alone (mRNA), whereas the total length was significantly reduced in both *itchb* MO-injected embryos (MoB) and embryos injected with a combination of *itchb* MO and *ITCH* mRNA (Rescue). Total length was significantly larger in the Rescue group than in the MoB group, indicating partial rescue. The number of embryos measured is indicated at the bottom of the graphs. Each graph summarizes at least four different experiments. 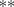 indicates 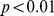.

We next examined the impact of loss of expression of *itch* genes in early development. We designed antisense splice junction morpholino oligonucleotides (MOs) targeting *itcha* and *itchb* (Table S2). We first assessed the efficacy of the MOs by performing RT-PCR on RNA extracted from control or injected embryos using oligonucleotide primers flanking the target exons of each MO. There was a marked and specific reduction of *itcha* mRNA in embryos injected with MOs against *itcha* but not *itchb*, and likewise for *itchb* with the *itchb* but not the *itcha* MO, with amplification of the lower, exon-skipped band readily visible in the middle panel (Fig. 3 A). Both MOs induced considerable cell death, visualized by acridine orange staining [25] (Fig. S1). Injection of morpholino has been shown to induce cell death by activating the proapoptotic protein p53, an unspecific side effect efficiently counteracted by downregulation of p53 expression [26]. Therefore, p53 MO [27] was consistently co-injected to reduce the general toxicity effect of MOs.

We then assessed the morphology of injected embryos. Knockdown of either gene caused a general growth delay, the effect of *itchb* knockdown being generally more severe (Fig. 3 B-I and K-N). *itchb* knockdown embryos were about two hours late in their development at 24 hpf, judging from the number of somites and pLL primordium migration. They remained smaller throughout the first five days of development, the discrepancy in size tending to increase with time (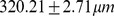 at 72 hpf compared to 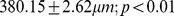) (Fig. 3 J,O). Other general features included small head, deformed hindbrain, small eyes, immature fin buds at 72 hpf, pericardium oedema and low survival after 96 hpf. They were able to swim but were generally not responsive to touch and tended to rest on the bottom of the dish when undisturbed (not shown). In comparison, *itcha* knockdown was less severe, but knockdown embryos were still significantly smaller than control siblings (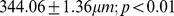) (Fig. 3 L–O). The coinjection of both MOs resulted in slightly smaller embryos 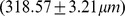 and high mortality (Fig. 3 N,O).

In an attempt to rescue and assess the specificity of the more severe *itchb* phenotype, we used *in vitro*-transcribed human *ITCH* mRNA (that is not targeted by the *itchb* MOs), injected alone as a control, or in *itchb* knockdown embryos. Co-injection of *ITCH* mRNA partially rescued growth defects, with embryos reaching a significantly larger size than upon *itchb* knockdown 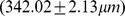, and was comparable to *itcha* knockdown (Fig. 3 P).

### *itchb* is involved in pLL development

**Figure 4:**
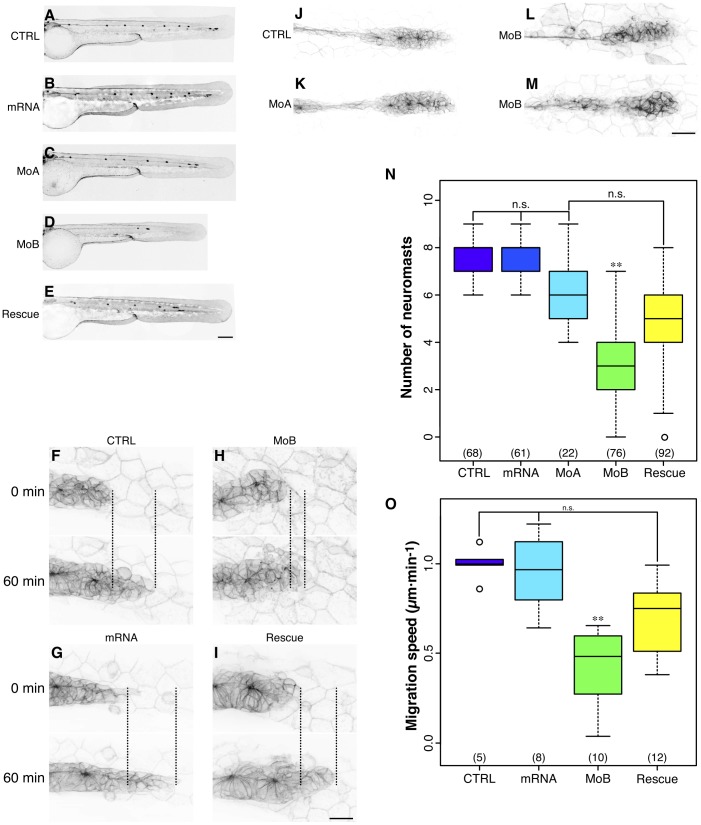
*itchb* is involved in pLL primordium migration and lateral line development. (A–E) Posterior lateral line at 48 hpf *cldnb:gfp* in embryos injected with vehicle (CTRL), *in vitro*-transcribed human *ITCH* mRNA (mRNA), MOs against *itcha* (MoA), MOs against *itchb* (MoB) or MOs against *itchb* and *in vitro*-transcribed human *ITCH* mRNA (Rescue). There was a marked reduction in the number of neuromasts in the pLL after *itchb* knockdown (D), but not in *itcha* knockdown, although the primordium had not yet reached the end of the tail at this time point (C). The *itchb* knockdown effect was partially rescued by injection of human *ITCH* mRNA (E). (F–I) Time-lapse confocal microscopy on 26–30 hpf *cldnb:gfp* embryos showed that the pLL primordium migration was slowed in *itchb* knockdown embryos (MoB) compared to vehicle (CTRL), human *ITCH* mRNA-injected (mRNA) or rescued embryos (Movies S1–S4). (N) The number of neuromasts in the posterior lateral-line was counted after staining with DiAsp in 72 hpf WT embryos injected with vehicle (CTRL), *in vitro*-transcribed human *ITCH* mRNA (mRNA), MOs against *itcha* (MoA), MOs against *itchb* (MoB) or MOs against *itchb* and *in vitro*-transcribed human *ITCH* mRNA (Rescue). Whereas the number of neuromasts in the pLL was markedly reduced after injection of MOs against *itchb*, injection of *in vitro* transcribed *ITCH* mRNA alone or of MOs against *itcha* had no effect. The MOs phenotype was rescued by co-injection of *ITCH* mRNA in *itchb* knockdown. The number of embryos for each group is indicated at the bottom of the graph. 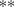 represents statistical significance at 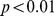. n.s.  =  non-significant. (J–M) Morphology of the migrating primordium in 26 hpf embryos (CTRL), *itcha* knockdown embryos (MoA) and *itchb* knockdown embryos (MoB). Cells at the tip of the primordium of MoB embryos were slightly disorganized, but the typical rosette formation occurred normally. (O) Migration of the pLL primordium was quantified by measuring the distance between the primordium leading edge in the first frame (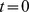) compared to the last frame (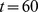) as shown in F–I (doted line). The number of embryos counted or imaged is indicated at the bottom of the graph. Each graph summarizes four different experiments. 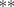
represents statistical significance at 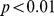. n.s. =  non-significant. EGFP fluorescence is presented as reversed black and white to facilitate visualization in all micrographs. Scale bars: (A–E) 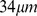, (F–I) 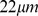, (L–M) 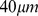.

In mammalian cells, Itch has been identified as an important regulator of CXCR4 signaling [14], [16]. This cytokine receptor is well described in Zebrafish for its role in pLL primordium migration [28]. We therefore examined the consequences of itch knockdown on pLL development. We used DiAsp, a vital marker of neuromast hair cells to label the pLL of WT embryos [29]. We also used *cldnb:gfp* embryos that express GFP in the developing pLL primordium as well as the neuromasts [30]. Both methods yielded identical results and showed that the pLL primordium did not reach the tail tip upon *itchb* knockdown, usually stalling around the end of the digestive track at 48–52 hpf (Fig. 4 D). In *itcha* knockdown, the primordium migration was slightly delayed at 48 hpf(Fig. 4 C), but had reached the tail by 52 hpf (not shown). The number of neuromasts formed at 72 hpf was drastically reduced in *itchb* knockdown, but not in *itcha* knockdown (Fig. 4 C, E and N).

The primordium maintained its overall morphology, and the characteristic rosette-like organization of the neuromast progenitors was readily identifiable under all conditions, but *itchb* morphants were often more disorganized and seemed to collapse in the trailing end (Fig. 4 J–M). They also often exhibited less cohesion in the border cells, as can be seen in Movie S3.

To determine if primordium migration was affected by ablation of *itchb*, we performed time-lapse microscopy experiments in *cldnb:gfp* embryos. We recorded primordium migration starting just before deposition of the first trunk neuromast in control, morphant or rescued embryos (Fig. 4 F–I and Movies S1–S4). Primordium migration was significantly slower in *itchb* morphants (Fig. 4 O and Movie S3), as compared to control (Fig. 4 O and Movies S1–S2). Moreover, cells from the primordium leading edge in morphant embryos were disorganized and moved around and back instead of straight toward the tail as in control primordia. This cell behavior suggests that the slowing of pLL primordium migration is not merely the consequence of general growth failure, but due to genuine interference with directional cell migration. The primordium migration defect was rescued by human *ITCH* mRNA injection (Fig. 4 I and Movie S4). Overall, the migration speed of *itchb* morphant primordia was reduced by approximately 50% (median speed 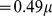
m

min^–1^ *vs* 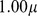
m

min^–1^ in controls) and 24% in rescue (median speed 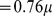
m

min^–1^) (Fig. 4 O).

### *itchb* knockdown perturbs signaling events required for pLL primordium migration

**Figure 5:**
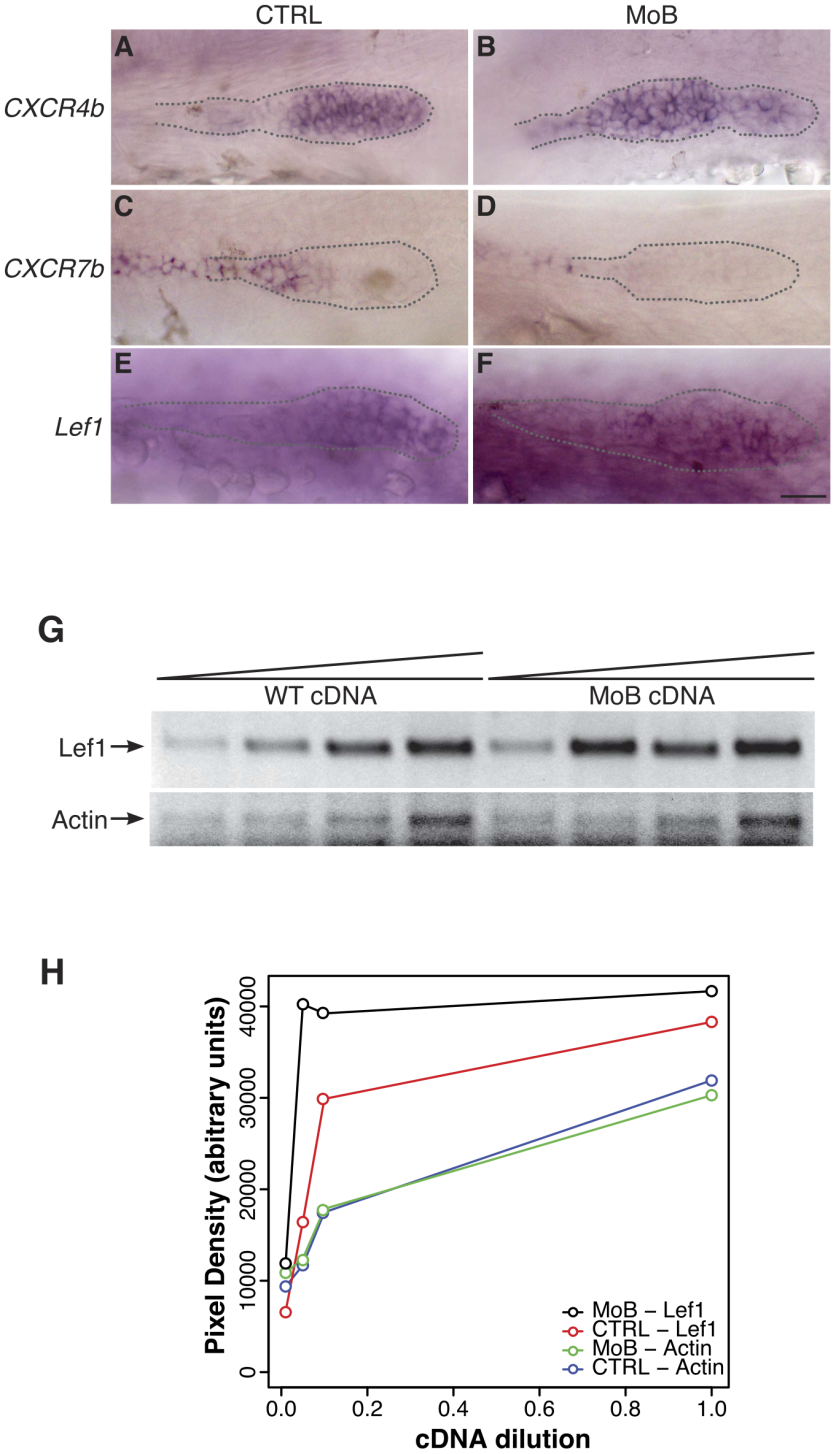
Primordium patterning is altered in *itchb* knocked-down embryos. (A–F) RNA *in situ* hybridization of factors required for primordium patterning in control embryos (vehicle-injected siblings) (CTRL, left panels) and MOs-injected embryos (MoB, right panels) at 30 hpf. *cldnb:gfp* embryos were used to ensure that the primordium had migrated pass the 10th somite before fixation. (A,B) *cxcr4b* expression was limited to the leading half of the primordium in control embryos, but extended throughout the primordium after *itchb* knockdown. (C,D) *cxcr7b* was limited to the trailing end of control embryos, and almost completely excluded from the primordium in *itchb* knockdown. (E,F) *lef1* was expressed in the leading edge of control embryos. Darker staining indicated higher expression in embryos injected with MOs against *itchb* (MoB), but leading edge expression was maintained. Scale bar: 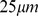. (G) Semi-quantitative RT-PCR showing enhanced expression of *lef1* in embryos injected with MOs against *itchb* (MoB) as compared to control embryos (WT). Amplification of the actin gene was used as an internal control. The image was inverted to facilitate quantification. (H) Densitometry measurements from the gel presented in G. This is representative of three different experiments.

Migration of the primordium along the myoseptum is directed by the cytokine receptor Cxcr4b, expressed in the leading edge of the primordium. Directionality is ensured by expression of another cytokine receptor, Cxcr7b, in the trailing end of the migrating primordium, whose expression is restricted by Cxcr4b and acts as a sink for the ligand Sdf-1, thereby preventing Cxcr4b signaling in the trailing end and allowing directionality of migration [31]. Given the slow migration of the pLL primordium in *itchb* morphants, we examined *cxcr4b* and *cxcr7b* expression in the primordium. We visually assessed in *cldnb:gfp* embryos that the primordium had reached somite 10 before fixation and *in situ* hybridization. This occurred at about 28 hpf for control embryos and 30 hpf for *itchb* morphants. Comparing *cxcr4b* expression in these stage-matched embryos, while *cxcr4b* expression was restricted to the leading two-third of the primordium in control embryos (Fig. 5 A), it was clear that *cxcr4b* was overexpressed in *itchb* morphants and that its distribution encompassed the entire primordium, extending to the deposited cells behind the migrating primordium (Fig. 5 A and B; representative of 11 embryos in 4 different experiments).

Reciprocally, *cxcr7b* expression, normally present in the trailing end and in the deposited cells in control embryos (Fig. 5 C) was almost completely excluded from the primordium of *itchb* morphants, and only visible in deposited cells further behind the primordium (Fig. 5 D; representative of 11 embryos from 3 different experiments). This pattern was consistent with the slow primordium migration measured in Figure 4 H and O.

### Wnt signaling is increased in *itchb* morphants

Wnt signaling also plays an important role in pLL primordium migration and is thought to act upstream of *cxcr4* [7]. Wei *et al.* [17] identified Disheveled, an important intermediate in the Wnt signaling pathway as a target of Itch in mammalian cells. To determine if Wnt signaling was modified by *itchb* knockdown, we examined the expression of the Wnt target gene *lef1*, whose expression is normally limited to the leading cells of the migrating primordium [7], as seen in control embryos (Fig. 5 E). In *itchb* morphants *lef1* expression appeared to be increased, though it was still restricted to the leading two thirds of the structure (Fig. 5 F).

To get a sense of the general level of *lef1* expression in *itchb* morphants, we performed a semi-quantitative RT-PCR experiment with RNA extracted from stage-matched control (WT) or *itchb* morphant (MoB) embryos. After reverse transcription, we performed serial dilutions of the cDNA reactions and proceeded to PCR amplification with specific primers for *lef1* and *actin* (Table S3). Amplification of *actin* was comparable between the two sets of cDNAs (Fig. 5 G), showing comparable extraction and reverse-transcription efficacy. In contrast, *lef1* was amplified from much lower amounts of cDNAs from *itchb* morphants than from control embryos, indicating higher expression of *lef1* in this group (Fig. 5 G, H). These data are consistent with general Wnt signaling activation in *itchb* morphants.

## Discussion

### *itchb* is required to maintain primordium migration

This study aimed at exploring how early vertebrate development was affected by knockdown of the ubiquitin ligase *itch* gene using the vertebrate model organism *Danio rerio*. We found two genes coding for proteins with similar sequence identity with human *ITCH*, *itcha* and *itchb*. RT-PCR experiments demonstrate that both genes were expressed in zebrafish larvae, and a generalized pattern of expression was observed using *in situ* hybridization. Duplicated genes are common in zebrafish and in these cases paralogues have been found to present partial genetic redundancy, although they also often diverge in either their function or their expression pattern [32]. Though knockdown of either or both genes affected overall growth, *itcha* and *itchb* indeed appeared to differ in their function, the most noticeable distinction being in the effect of the knockdown of *itchb* but not *itcha* on perturbation of pLL development.

The process governing pLL primordium migration is well described and involves Wnt, Fgf and cytokine signaling. The principal determinant of pLL primordium migration is the establishment of distinct expression domains of the cytokine receptors *cxcr4b* and *cxcr7b*. As the primordium migrates along the myoseptum, *cxcr4b* is restricted to the leading half of the primordium and inhibits *cxcr7b*, which consequently is present only in the trailing end [1], [7], [33]. This asymmetric distribution directs primordium migration along the myoseptum in response to Sdf1 secretion and disturbing this equilibrium results in slowed primordium migration speed or stalling of the primordium [1], [7], [33]–[35]. Wnt signaling occurs mainly in the leading region of the pLL primordium and activates Fgf signaling in the medial and trailing region [7]. Fgf signaling organizes the primordium precursor cells in rosettes that will become the neuromasts and restricts Wnt signaling to the leading cells [36], [37].

The expression of *cxcr4b* is regulated at the transcriptional level by Wnt and oestrogen signaling, as response elements for Lef1 and Esr1 are present in the upstream control region of the *cxcr4b* gene [34], [35]. *lef1* morphants and mutants demonstrate truncated pLL, as the migrating primordium collapses before it reaches the end of the tail caused by decreased cell proliferation and lack of progenitors [4], [35], [38]. Lef1 depletion alone has no effect on *cxcr4b* or *cxcr7b* expression, and pLL primordium migration and differentiation appears to be normal. However, increasing Wnt signaling, as occurs in *apc* mutants, strongly inhibits primordium migration while increasing *cxcr4b* expression domain and excluding *cxcr7b* from the primordium [7].

*itchb* morphants similarly exhibited displacement of the *cxcr4b* and *cxcr7b* expression domains, consistent with increased *cxcr4b* and Wnt signaling. In cultured mammalian cell lines, Itch has been shown to increase both Wnt and Cxcr4 signaling. Itch can regulate the Wnt signaling pathway by recognizing and ubiquitylating phosphorylated Dvl [17]. Dvl is recruited to the activated Wnt receptor complex and activates both the canonical and non-canonical signaling pathways [19]. Upon activation by Wnt, Dvl become hyperphosphorylated, and this phosphorylation is essential to fully activate 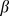-catenin stabilization [39], [40]. Inactivation of Itch stabilizes phosphorylated Dvl, increasing Wnt signaling [17]. In zebrafish, Dvl degradation has been shown to be implicated in Wnt signaling regulation [41]. On the other hand, Dvl expression has also been shown to be stable in zebrafish embryos during primordium migration [42]. It must be stressed that Itch specifically targets phosphorylated Dvl and promotes its proteasomal degradation [17]. Consequently, Itch depletion could increase Wnt signaling and expression of Wnt signaling pathway target genes, without affecting the overall level of Dvl protein [17].

CXCR4 is a direct target of ITCH ubiquitin ligase activity in mammalian cells [14], [15]. ITCH is known to regulate CXCR4 internalization and signaling in conjunction with 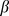-arestin and STAM [16], [43]. CXCR4 ubiquitylation is required for its rapid ligand-induced lysosomal degradation [15]. ITCH interacts directly with CXCR4 and HRS to direct CXCR4 degradation [14], [44]. Moreover, ITCH is necessary for CXCR4-mediated activation of the ERK/MAPK pathway [16]. It has not been possible to directly measure the effect of Itch depletion on Cxcr4b protein, as antibodies against CXCR4 do not discriminate Cxcr4a and Cxcr4b in Western blot analysis and did not yield a reliable signal in immunofluorescence. Nevertheless, *cxcr4b* is clearly overexpressed in the primordium at the mRNA level, which could be a direct effect of its increased signaling. It is known that Sdf1/Cxcr4 signaling exerts a positive feedback on *cxcr4b* expression in the primordium [1], [34]. Therefore, increased *cxcr4b* expression is consistent with Cxcr4b protein stabilization. Cxcr4b also exerts transcriptional control over *cxcr7b* expression, effectively excluding Cxcr7b form the Cxcr4b expression domain [1]. This is consistent with the reduced *cxcr7b* signal in *itchb* morphants.

Defects in pLL primordium migration in *itchb* morphants can thus be attributed to increased Wnt and Cxcr4b signaling *in vivo*. Since the phenotypic manifestation of perturbed Cxcr4b signaling and Wnt overactivation are confounded, it is not possible to discriminate which pathway is most affected by *itchb* depletion, though most likely *itchb* affects both.

### Neuromast deposition is altered in zebrafish depleted of itchb

Although neuromast deposition is clearly a consequence of and influenced by pLL primordium migration, manipulations specifically altering neuromast deposition have no effect on pLL primordium migration [45]. The processes are thus independently regulated by overlapping signals. Examining the pLL of *itchb* deficient embryos, it was clear that both the number of neuromasts and the rate of primordium migration were reduced by half. Reduced neuromast deposition is observed when the proliferation rate is decreased, for example by treating the embryos with DNA replication inhibitors such as hydroxyurea [45]. Increased cell death also leads to fewer neuromast deposition, as seen in *bap28* homozygous mutants and *tcf7* ATG morphants. Importantly, the reduction in proneuromast number in *tcf7* MO injected embryos is non-specific and not due to loss of *tcf7* function, and was completely rescued by the coinjection of *p53* MOs [45]. Both *itcha* and *itchb* MOs indeed induced significant cell death when injected alone, but this effect was reversed by addition of p53 MO. Moreover, only *itchb* morphants exhibited defects in neuromast deposition, and this effect was rescued by injection of human Itch mRNA. p53-mediated cell-death therefore is unlikely to be responsible for the neuromast deposition defects. Nevertheless, reduction of Itch has been related to increased cell death or reduced proliferation in a number of cases. First, p63 and p73, two isoforms of p53, are direct targets of Itch, and their stabilization through Itch depletion is known to activate apoptotic pathways [46]–[48]. Although it has not been reported specifically in the primordium, p63-mediated cell death occurs in zebrafish embryos and larvae [49]. In mammalian cells, ITCH depletion increased LATS1, a serine/threonine kinase in the Hippo pathway, enhancing FAS-induced apoptosis and reducing proliferation, survival, and migration [50]. This is consistent with results showing that in HEK-293T cells, ITCH depletion decreased cell survival and enhanced TRAIL-induced cell-death [51]. Increased cell death is thus a plausible explanation for loss of neuromasts in *itchb* morphants, but its assessment is difficult due to the recognized caveat in the use of MOs to examine apoptotic pathways [26].

It must be noted that Itch depletion has also been shown to induce cell proliferation through a number of signaling pathways. In hematopoietic stem cells, Itch deficiency increases proliferation by stabilizing Notch1 signaling [52]. In the zebrafish pLL, Notch signaling determines differentiation of pLL ganglion neurons and, later on, of hair cells [53], [54]. Although it was shown that preventing hair cell differentiation leads to fewer neuromasts in the pLL [55], the effect of promoting Notch signaling on this system is unknown. Moreover, since DiAsp was successfully accumulated by those neuromasts that were formed, we conclude that *itchb* morphant hair cells were functional. In the context of Hedgehog signaling, Itch depletion promoted tumorigenicity, preventing the formation of a degradation complex between Itch, Numb and Gli1. This had the effect of stabilizing Gli1 and promoting medulloblastoma growth [56]. There is thus a multitude of pathways through which Itch could influence both cell-death and cell proliferation in the pLL primordium that could result in the formation of fewer neuromasts. A transcriptomic or proteomic approach could yield knowledge about how *Itchb* affects neuromast formation.

In the zebrafish pLL primordium, proliferation is regulated mainly by Wnt and Fgf signaling, and seems to be largely independent of cell differentiation [45]. Increasing Wnt signaling alone leads to increased proliferation, but the process is dependent on Fgf signaling [45], [57]. In short, Fgf signaling promotes proliferation whereas Wnt signaling limits the proliferation zone to the trailing end of the primordium, where rosette formation occurs, while there is little proliferation in the leading zone that instead directs migration [45]. The domain of *cxcr7b* expression seems to delimit the zone where the depositing cells reside, although it is not clear how Cxcr7b activity mediates this process [45]. *cxcr7b* expression is limited by Wnt signaling and *lef1* expression that occurs in the leading zone of the primordium [7]. *lef1* is a target gene of Wnt signaling and its expression increases in *apc* mutants [7]. We show here that Itch depletion in zebrafish resulted in increased Wnt signaling, as was shown by higher *lef1* expression, consistent with the identification of Dvl as a target of Itch ubiquitylation in mammalian cells [17].

Increased Wnt signaling in zebrafish is associated with slower migration of the primordium, but is not sufficient to induce deposition of fewer neuromasts. Apart from increased apoptosis, the reduction in neuromast number in *itchb* morphants could occur through altered Fgf signaling [45], [58]. No direct impact of Itch on Fgf signaling has been reported so far in the literature. Fgfr1 is a direct target of the closely related Nedd4-1 ubiquitin ligase and the inhibition of this interaction leads to an important increase in Fgf signaling that alters anterior development in zebrafish [59]. Ubiquitylation is important to regulate Fgfr internalization and signaling, both directly and indirectly through the regulation of Sprouty 2 [59], [60]. Tyrosine kinase receptor internalization is influenced by ubiquitylation of the endocytic machinery, and targets of Itch have been implicated in this process [10]–[12], [61]–[65]. However, should Itch be directly involved in Fgf regulation, one would expect that *itchb* depletion would lead to increased Fgf signaling. Increasing Fgf signaling does not lead to reduced proliferation, but instead leads to the formation of supplementary rosettes in the migrating primordium [37]. It does not seem likely then that the Fgf pathway is perturbed independently of Wnt and Cxcr4b in *itchb* morphants.

The HECT-domain ubiquitin ligase Itch is an important negative regulator of signaling. It has been mainly linked to immunological responses, but is widely expressed and likely involved in many developmental and regulatory signaling events.

In mice, Itch deficiency results in spontaneous development of late onset and progressively lethal systemic autoimmune-like disease, attributable to biased differentiation of CD4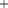 cells into T_H_2 cells and chronic activation [66]. The inflammatory response is also attributable to expansion of the B1b lymphocytes leading to IgM elevation and IgE production [67]. These immunological defects are mainly attributable to accumulation of the transcription factor JunB, in the absence of Itch [66], [67].

In human, in addition to multisystem autoimmune diseases akin to the Itchy mice phenotype, patients with *ITCH* mutations displayed morphologic and developmental abnormalities [68]. Together, the results described above confirm that Itch constitutes an important signaling hub in the cell, maintaining the balance in several important signaling pathways. Different vertebrate models, including the zebrafish introduced here, are likely to unveil the molecular defects underlying these Itch-related pathologies.

## Materials and Methods

### Ethics Statement and Transgenic Animals

A colony of wild-type Longfin zebrafish (*Danio rerio*) was bred and maintained according to standard procedures in our animal facility [69]. The transgenic line Tg(*cldnb:lynEGFP*) expressing membrane-tethered EGFP (enhanced GFP) under the *claudinb* promoter was used as it labels the migrating lateral line primordial, the neuromast organs as well as the chain of interneuromast cells deposited during migration [30]. All experiments were performed in compliance with the guidelines of the Canadian Council for Animal Care and approved by the *Comité de déontologie de l'expérimentation sur les animaux* (CDEA) of the University of Montreal. Embryos were anesthetized in 0.02% tricain (MS-222, Sigma) in Embryo medium prior to all experiments.

### Antisense Morpholino Oligonucleotides and RNA Injections

To knockdown the zebrafish *itcha* and *itchb* genes, we designed splice-junction blocking MOs specific to the donor and acceptor splice-sites of *itcha* and *itchb* exons 12 and 13 (Gene Tools, Philomath, OR). MOs and mRNAs were diluted in nuclease-free water with 0.2% FastGreen vital dye to judge of injection volume. To avoid toxicity effects, p53 MO was coinjected [27]. All MOs are listed in the file (Table S2).

Human *ITCH* mRNA was transcribed from the I.M.A.G.E. Consortium (LLNL) cDNA Clone 4838366 [70] encoding human *ITCH* linearized with BamHI using the mMESSAGE Machine T7 kit (Ambion, Austin, TX).

### Reverse Transcription-PCR

Total RNA was extracted from pools of approximately 50 embryos, treated as stated, using TRIzol Reagent (Invitrogen, Carlsbad, CA). cDNA was synthesized from 1 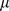
g total RNA using the SuperScript VILO cDNA Synthesis Kit (Invitrogen, Carlsbad, CA). All amplifications were carried on using Phusion High Fidelity DNA polymerase (New England Biolab, Ipswich, MA).

Semi-quantitative PCR reactions were setup using serial dilutions of cDNAs followed by 30 cycles of amplification. All primers are listed in the file (Table S3).

### Lateral Line Staining

The lateral line of control or injected embryos was labeled using the vital dye 4-(4-diethylaminostyryl)-N-methylpyridinium iodine (4-di-2-ASP, Invitrogen) diluted to 0.5 mM in embryo medium according to an established procedure [71]. Embryos were dechorionated manually and staged at 72 hpf then incubated in the solution for 30 minutes at 28.5°C. They were then washed three times for 10 minutes in fresh embryo medium and anesthetized in tricain before imaging on an epifluorescence dissection microscope (Olympus) equipped with a Flea2 CCD Camera (IEEE 1394, Point Grey Research Inc. Richmond, BC, Canada). This protocol allows for visualization of full neuromasts that were counted for each fish.

### Acridine Orange Staining

Zebrafish were incubated in 1 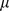
g/ml acridine orange for 30 min then repeatedly washed in embryo medium. Larvae were anesthetized in tricain and mounted in low melting point agarose before being visualized under a 10X water immersion lens mounted on a Quorum Technologies spinning disk confocal microscope as bellow.

### Confocal Microscopy and Time-Lapse Recordings

Embryos were anesthetized in 0.02% tricain (MS-222) in embryo medium and embedded in 1% low melting point agarose. Imaging was performed on a Quorum Technologies spinning-disk confocal microscope (Quorum WaveX Technology Inc Guelph, On, Canada) mounted on an upright Olympus BX61W1 fluorescence microscope with water-immersion lenses. The setup was fitted with a Hamamatsu ORCA-ER camera and image acquisition was done with the Volocity software (Perkin-Elmer) and analyzed with the ImageJ software (NIH). Stacks were acquired at 1 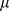
m thickness and assembled in ImageJ. When necessary, adjacent frames were aligned and stitched together using Photoshop CS 5.1 (Adobe Systems Incorporated) Auto-Blend Layers function. For time-lapse microscopy, images were captured every three minutes for 60 minutes. To quantify the primordium migration, the distance between the tip of the primordium at 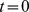 and the tip of the primordium at 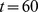 minutes was measured in ImageJ after superposition of the images.

### Whole-Mount *In Situ* Hybridization

*In situ* hybridization was performed using sense and antisense probes designed against the zebrafish orthologs of *itch* (Table S4) to view endogenous localization of *itch* mRNA. Embryos of 6 hpf and 24 hpf were processed for *in situ* hybridization as previously described [72]. To measure the impact of *itch* down-regulation on signaling in the migrating primordium, probes against *cxcr4b*, *cxcr7b* and *lef1* (Table S4), were synthesized and used in an identical procedure on MO and control injected *cldnb:gfp* embryos age-staged at 26 hpf according to primordium migration.

## Supporting Information

Figure S1
**Cell death in the pLL primordium of** ***itcha*** **and** ***itchb*** **morphants.** (A–C), acridine orange staining in the primordium region of control *cldnb:gfp* embryos (CTRL), *itcha* knockdown (MoA), and *itchb* knockdown (MoB). p53 MO was omitted in this experiment. In these conditions, cell death occurred in the primordium cells, predominantly in the trailing end of the migrating primordium in both *itcha* (B) and *itchb* (C) morphants. Acridine orange staining is visible as brighter dots representing the nucleus of apoptotic cells over the dimmer EGFP signal in the cell membrane of the transgenic primordium. Scale bar: 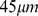.(TIF)

Table S1 **Accession number of sequences used for alignment.**
(PDF)

Table S2 **Sequence of morpholino oligonucleotides.**
(PDF)

Table S3 **Sequence of the PCR primers used in this study.**
(PDF)

Table S4 **cDNA regions used as probes in in situ hybridization experiments.**
(PDF)

Movie S1
**CTRL.** Example of a pLL primordium migration at 28 hpf in a control embryo. Stacks were acquired at 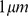 thickness and assembled in ImageJ. Movie length: 60 min.(AVI)

Movie S2
**mRNA.** Example of a pLL primordium migration at 28 hpf in a human *ITCH*-injected embryo (a second control). Stacks were acquired at 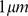 thickness and assembled in ImageJ. Movie length: 60 min.(AVI)

Movie S3
**MoB.** Example of a pLL primordium failing to migrate at 30 hpf in a *itchb*-morpholino-injected embryo. Stacks were acquired at 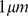 thickness and assembled in ImageJ. Movie length: 60 min.(AVI)

Movie S4
**Rescue.** Example of a pLL primordium migration at 28 hpf in a *itchb*-morpholino-injected embryo after rescue with human *ITCH* mRNA. Stacks were acquired at 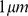 thickness and assembled in ImageJ. Movie length: 60 min.(AVI)
