# Supplementary figures and images for: Itch Is Required for Lateral Line Development in Zebrafish
Source: PLoS One. 2014 Nov 4;9(11):e111799. doi: 10.1371/journal.pone.0111799 (PMC4219781; doi:10.1371/journal.pone.0111799)

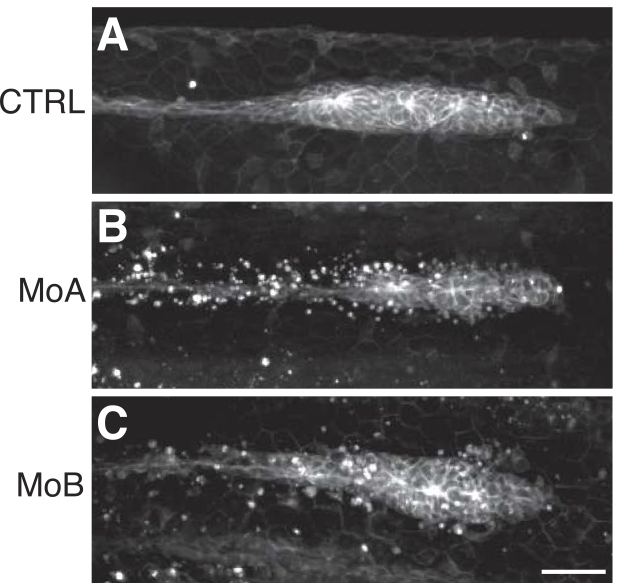

Supplement: Figure S1 — Cell death in the pLL primordium of itcha and itchb morphants. (A–C), acridine orange staining in the primordium region of control cldnb:gfp embryos (CTRL), itcha knockdown (MoA), and itchb knockdown (MoB). p53 MO was omitted in this experiment. In these conditions, cell death occurred in the primordium cells, predominantly in the trailing end of the migrating primordium in both itcha (B) and itchb (C) morphants. Acridine orange staining is visible as brighter dots representing the nucleus of apoptotic cells over the dimmer EGFP signal in the cell membrane of the transgenic primordium. Scale bar: . (TIF) [file pone.0111799.s001.tif]
