# Supplementary material for: Itch Is Required for Lateral Line Development in Zebrafish
Source: PLoS One. 2014 Nov 4;9(11):e111799. doi: 10.1371/journal.pone.0111799 (PMC4219781; doi:10.1371/journal.pone.0111799)
Supplement: Table S1 — Accession number of sequences used for alignment. (PDF) [file pone.0111799.s002.pdf]

Table S.1. Accession number of sequences used for alignment

Gene	Accession Number
Human	
ITCH	NM_001257137
WWP1	NM_007013
WWP2	NM_007014
NEDL2	NM_020760
NEDL1	NM_015052
NEDD4L	NM_001144967
NEDD4	NM_006154
SMURF2	NM_022739
SMURF1	NM_020429
Zebrafish	
itcha	XM_002667532
itchb	XM_003201654
wwp1	XM_003201365
wwp2	NM_001099448
nedl1	NM_001145764
nedl2a	XM_001922405
nedl2b	XM_003197612
nedd4l	XM_682997
nedd4	NM_001034186
smurf1	NM_001001943
smurf2	NM_001114426
Outgroup	
Monosiga brevicolis	XM_001743996
