# Supplementary material for: Itch Is Required for Lateral Line Development in Zebrafish
Source: PLoS One. 2014 Nov 4;9(11):e111799. doi: 10.1371/journal.pone.0111799 (PMC4219781; doi:10.1371/journal.pone.0111799)
Supplement: Table S2 — Sequence of morpholino oligonucleotides. (PDF) [file pone.0111799.s003.pdf]

Table S.2. Sequence of morpholino oligonucleotides

Name	Target	Sequence	Type	Binds to
A1a	*itcha*	tgcagctcataatgatcccacCCTT[a]	Splice	Exon 12 3' end
A3b	*itcha*	ataatataagaactcacAGCGAGGA	Splice	Exon 13 3' end
B4b	*itchb*	atgtgtttgcactcacAGGGAGGAT	Splice	Exon 13 3' end
tp53[b]	p53	GCGCCATTGCTTTGCAAGAATTG	Translation	Start codon

[a]Lowercase letters are complementary to intron sequence.
[b]Langheinrich *et al.* [27].
