# Supplementary material for: Itch Is Required for Lateral Line Development in Zebrafish
Source: PLoS One. 2014 Nov 4;9(11):e111799. doi: 10.1371/journal.pone.0111799 (PMC4219781; doi:10.1371/journal.pone.0111799)
Supplement: Table S3 — Sequence of the PCR primers used in this study. (PDF) [file pone.0111799.s004.pdf]

**Table S.3. Sequence of the PCR primers used in this study**

| Name | Sequence | Position |
|---|---|---|
| *itcha*-2F | 5'-CAGGAAAATCCTCGCTTGAG-3' | 1550–1569 |
| *itcha*-2R | 5'-GTGGATGGCAACCCATAATC-3' | 1710–1729 |
| *itcha*-8F | 5'-GCGCCGACAGCTAATAAAGA-3' | 1297–1316 |
| *itcha*-8R | 5'-CTCAAGCGAGGATTTTCCTG-3' | 1550–1569 |
| *itchb*-1F | 5'-GAACCCGATGTACTGCCTGT-3' | 1859–1878 |
| *itchb*-1R | 5'-TGTTCAGGATGCGCTTGTAG-3' | 2033–2052 |
| *itchb*-4F | 5'-GCTTCAGCCACCCAGAATAA-3' | 1320–1339 |
| *itchb*-3R | 5'-AAAGAGAGTTTTGCGGCTGA-3' | 1699–1718 |
| *cxcr4b*-F | 5'-GCCACAAACAGCCAAAACTT-3' | 461–480 |
| *cxcr4b*-R | 5'-CAGCATAGTCAAAGCGTCCA-3' | 837–856 |
| *cxcr7b*-F | 5'-AGAGCCGAGAGGACACGTTA-3' | 344–363 |
| *cxcr7b*-R | 5'-GGCGTCTGAATGGTCAGATT-3' | 693–712 |
| *actin*-F | 5'-AAGGCCAACAGGGAAAAGAT-3' | 434–453 |
| *actin*-R | 5'-GGTACGACCGGAGGCATAC-3' | 523–541 |
| *gapdh*-F | 5'-CTTGGCTCCTCTGGCTAAAGTT-3' | 522–543 |
| *gapdh*-R | 5'-GTCATACCAGGAGATGAGCTTGAC-3' | 976–999 |
