# Supplementary material for: Itch Is Required for Lateral Line Development in Zebrafish
Source: PLoS One. 2014 Nov 4;9(11):e111799. doi: 10.1371/journal.pone.0111799 (PMC4219781; doi:10.1371/journal.pone.0111799)
Supplement: Table S4 — cDNA regions used as probes in in situ hybridization experiments. (PDF) [file pone.0111799.s005.pdf]

Table S.4. cDNA regions used as probes in in situ hybridization experiments

**Target**	**Reference**	**Fragment**	**Vector**
itcha	XM_002667532	1297–1729	pCRII
itchb	XM_003201654	1320–2052	pCRII
cxcr4b	NM_131834	461–856	pCR4Blunt
cxcr7b	NM_001083832	344–712	pCRII
lef1	NM_131426	148–939	pCRII
